# Diagnostic Accuracy of Ambulatory Devices in Detecting Atrial Fibrillation: Systematic Review and Meta-analysis

**DOI:** 10.2196/26167

**Published:** 2021-04-09

**Authors:** Tien Yun Yang, Li Huang, Shwetambara Malwade, Chien-Yi Hsu, Yang Ching Chen

**Affiliations:** 1 School of Medicine College of Medicine Taipei Medical University Taipei Taiwan; 2 Department of Family Medicine Taipei Medical University Hospital Taipei City Taiwan; 3 International Center for Health Information Technology Taipei Medical University Taipei Taiwan; 4 Division of Cardiology, Department of Internal Medicine School of Medicine, College of Medicine Taipei Medical University Taipei Taiwan; 5 Division of Cardiology and Cardiovascular Research Center, Department of Internal Medicine Taipei Medical University Hospital Taipei Taiwan; 6 Department of Family Medicine School of Medicine, College of Medicine Taipei Medical University Taipei City Taiwan

**Keywords:** atrial fibrillation, ambulatory devices, electrocardiogram, photoplethysmography, diagnostic accuracy, ubiquitous health, mobile health, technology, ambulatory device

## Abstract

**Background:**

Atrial fibrillation (AF) is the most common cardiac arrhythmia worldwide. Early diagnosis of AF is crucial for preventing AF-related morbidity, mortality, and economic burden, yet the detection of the disease remains challenging. The 12-lead electrocardiogram (ECG) is the gold standard for the diagnosis of AF. Because of technological advances, ambulatory devices may serve as convenient screening tools for AF.

**Objective:**

The objective of this review was to investigate the diagnostic accuracy of 2 relatively new technologies used in ambulatory devices, non-12-lead ECG and photoplethysmography (PPG), in detecting AF. We performed a meta-analysis to evaluate the diagnostic accuracy of non-12-lead ECG and PPG compared to the reference standard, 12-lead ECG. We also conducted a subgroup analysis to assess the impact of study design and participant recruitment on diagnostic accuracy.

**Methods:**

This systematic review and meta-analysis was conducted in accordance with the Preferred Reporting Items for Systematic Reviews and Meta-Analysis (PRISMA) guidelines. MEDLINE and EMBASE were systematically searched for articles published from January 1, 2015 to January 23, 2021. A bivariate model was used to pool estimates of sensitivity, specificity, positive likelihood ratio (PLR), negative likelihood ratio (NLR), and area under the summary receiver operating curve (SROC) as the main diagnostic measures. Study quality was evaluated using the quality assessment of diagnostic accuracy studies (QUADAS-2) tool.

**Results:**

Our search resulted in 16 studies using either non-12-lead ECG or PPG for detecting AF, comprising 3217 participants and 7623 assessments. The pooled estimates of sensitivity, specificity, PLR, NLR, and diagnostic odds ratio for the detection of AF were 89.7% (95% CI 83.2%-93.9%), 95.7% (95% CI 92.0%-97.7%), 20.64 (95% CI 10.10-42.15), 0.11 (95% CI 0.06-0.19), and 224.75 (95% CI 70.10-720.56), respectively, for the automatic interpretation of non-12-lead ECG measurements and 94.7% (95% CI 93.3%-95.8%), 97.6% (95% CI 94.5%-99.0%), 35.51 (95% CI 18.19-69.31), 0.05 (95% CI 0.04-0.07), and 730.79 (95% CI 309.33-1726.49), respectively, for the automatic interpretation of PPG measurements.

**Conclusions:**

Both non-12-lead ECG and PPG offered high diagnostic accuracies for AF. Detection employing automatic analysis techniques may serve as a useful preliminary screening tool before administering a gold standard test, which generally requires competent physician analyses. Subgroup analysis indicated variations of sensitivity and specificity between studies that recruited low-risk and high-risk populations, warranting future validity tests in the general population.

**Trial Registration:**

PROSPERO International Prospective Register of Systematic Reviews CRD42020179937; https://www.crd.york.ac.uk/prospero/display_record.php?RecordID=179937

## Introduction

Atrial fibrillation (AF) is the most common cardiac arrhythmia worldwide, affecting approximately 33.5 million individuals. AF is more prevalent with increasing age, and its prevalence is expected to double by 2030 [1]. The disease can result in considerable morbidity and mortality by increasing the risk of heart failure, stroke, major cardiovascular events, sudden cardiac death, chronic kidney disease, peripheral arterial disease, and all-cause mortality despite often being asymptomatic [2]. A range of management choices, including anticoagulation, rate control, and rhythm control through medication or electrical cardioversion, can markedly reduce risks and relieve symptoms.

Although the treatment of AF is well established because of numerous guidelines and clinical trials, the detection of the disease remains challenging. The gold standard for AF detection is the 12-lead electrocardiogram (ECG) [3]. However, this method is not always available and can be unfeasible in certain groups of patients, such as individuals with paroxysmal AF who fail to undergo a 12-lead ECG in a timely manner or individuals with silent (subclinical) AF without any related symptoms. Therefore, devices that are convenient, inexpensive, and ambulatory are required to serve as preliminary screening tools; subsequently, initial diagnoses can be confirmed or excluded using the gold standard 12-lead ECG in hospital settings [4,5].

Several studies have investigated the accuracy and applicability of various ambulatory devices over the past few years. Rather than focusing on the devices themselves, this review targeted technologies used in ambulatory devices; thus, the summarized results are not limited to certain products. Among reviews of the use of ambulatory devices for AF detection, only one systematic review and meta-analysis focused on the accuracy of technologies compared with the gold standard [6]. That review concluded that blood pressure monitors and non-12-lead ECGs were most accurate; however, it failed to consider a newer technology, photoplethysmography (PPG). PPG is a new technology that has become ubiquitous in recent years, and one of its most widely known implementations is the Apple Watch [7]. Therefore, we conducted an updated systematic review focusing on 2 technologies that are used in ambulatory devices to detect AF: non-12-lead ECG and PPG. Additionally, the review focused on the automatic detection of AF utilizing built-in algorithms to validate their use as a convenient screening tool.

The aim of this paper was to provide a systematic overview of the accuracy of the 2 technologies compared with 12-lead ECG in the detection of AF as well as to describe their applicability, potential, and limitations.

## Methods

### Literature Search and Selection Criteria

**Figure 1:**
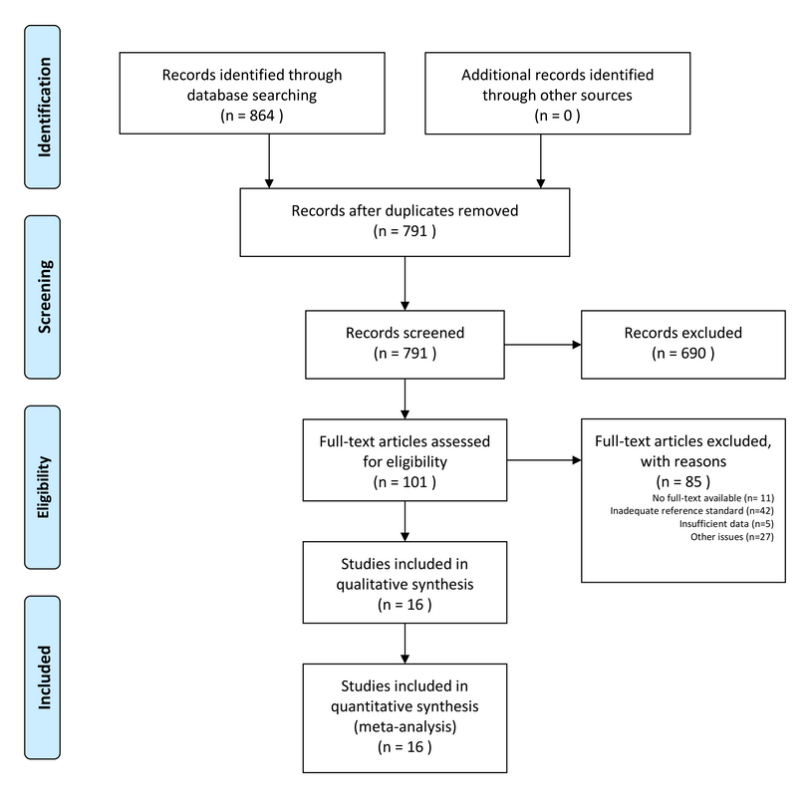
Summary of the study selection process using the PRISMA (Preferred Reporting Items for Systematic Reviews and Meta-Analyses) flow diagram.

We conducted a systematic search of MEDLINE (Ovid) and EMBASE for articles published from January 1, 2015 to January 23, 2021 using the search terms “mHealth,” “telemedicine,” “wearable,” “mobile health,” “mobile application,” and “digital treatment” in combination with the term “atrial fibrillation.” The search terms are presented in Multimedia Appendix 1. In addition, reference lists of relevant systematic reviews and included studies were hand-searched to identify additional articles. Only papers in English were included. All randomized trials, observational studies, and case series were included, whereas systematic reviews and case reports were excluded. Studies that recruited participants aged ≥18 years, investigated any method of identifying patients with suspected AF using an ambulatory device equipped with automatic interpretation by a mobile app or algorithm, provided a reference standard with 12-lead ECG interpreted by a competent professional, and reported sufficient data to enable the calculation of the diagnostic accuracy were included. Studies that investigated invasive methods of identifying AF, focused on the training of algorithms, validated the method and algorithm through a dataset, or failed to provide a timely reference standard for all participants were excluded. Two reviewers independently conducted the screening and reviewing of articles, and any disagreements were resolved by consensus with a third reviewer. The study strictly followed the PRISMA (Preferred Reporting Items for Systematic Reviews and Meta-Analyses) guidelines, and the PRISMA search flow diagram is provided in Figure 1.

### Data Extraction and Quality Assessment

Two reviewers independently extracted the data for the selected articles, including the year of publication, country, study design, number of study participants, average age of the study population, characteristics of the study population, technology used for measurement, measurement time, and reference standard. The absolute numbers of true positive, false positive, false negative, and true negative were also extracted.

If a study provided data on both the measurement level and individual level, the data analysis performed on the measurement level was extracted first. If a study provided both training data and validation data, only validation data were extracted. If a study failed to provide sufficient data for the calculation of diagnostic accuracy, the lead authors of the studies were contacted to request the missing data. If the authors failed to reply, 2 reviewers calculated the incomplete information on the basis of available data. Studies were excluded only if both the aforementioned methods failed to identify any additional data.

Study quality was evaluated using the quality assessment of diagnostic accuracy studies (QUADAS-2) tool, which is recommended for the evaluation of risk of bias and applicability in diagnostic accuracy studies [8,9]. The assessment tool focuses on 4 key domains: patient selection, index test, reference standard, and flow and timing. Risk of bias was evaluated in all 4 domains, and applicability concerns were evaluated in the first 3 domains. For each question, each study was graded as “low risk,” “high risk,” or “unclear risk.” A standardized table, recommended by the QUADAS-2 official website, was used to display the summarized results of the study quality appraisal.

### Statistical Analysis

Diagnostic accuracy statistics were computed using R software version 4.0.0 (R Core Team). The pooled sensitivity, specificity, positive likelihood ratio (PLR), negative likelihood ratio (NLR), and diagnostic odds ratio (DOR) — with their respective 95% CIs — were calculated using a bivariate diagnostic random-effects model in the *mada* package [10]. Tests for heterogeneity regarding the DOR were also performed and presented with Cochran Q and Higgins I^2^. Summary receiver operating characteristic (SROC) curves were used to summarize and visualize the diagnostic performance of each included study. The area under the summary receiver operating characteristic (AUSROC) curve was also calculated for both the non-12-lead ECG and PPG methods.

Publication bias was assessed using the Deek funnel plot asymmetry test, which is a scatterplot of (1/root [ESS]) against (ln[DOR]) [11]; a *P* value <.10 indicated publication bias. Fagan nomogram analysis was performed to determine the posttest probability of the disease based on the likelihood ratio of the diagnostic test. An age-adjusted prevalence of 0.5% was applied in the analysis to represent the general population worldwide [12], whereas a prevalence of 2.3% was adopted to represent a population with higher risks as targeted in a systematic review [13]. The left axis of a Fagan nomogram represents the pretest probability, the middle axis displays the likelihood ratio of the diagnostic test, and the right axis indicates the posttest probability. 

Because the risk of patient selection bias among the studies was obvious, a subgroup analysis between low-risk groups (studies that recruited patients with and without AF) and high-risk groups (studies that recruited only patients with AF) was conducted to investigate the effect of the study design and population on diagnostic performance. Data synthesis and most statistical analyses were conducted using R version 4.0.0.

### Study Registration

This systematic review and meta-analysis was registered in the PROSPERO International Prospective Register of Systematic Reviews (ID: CRD42020179937).

## Results

### Literature Search 

Figure 1 illustrates the flowchart of study inclusion. The initial search yielded 864 publications from MEDLINE (Ovid) and EMBASE. Duplicates and irrelevant studies were removed, yielding 791 publications for title and abstract review. After exclusion of 690 publications for irrelevant study focus, the rest of the 101 publications were then assessed through full-text articles. In total, 85 publications were excluded for the following reasons: having insufficient context, being an inappropriate study type, having a study population aged <18 years, utilizing invasive or implantable devices to identify AF, or having an inadequate reference standard. The full-text evaluation yielded 16 publications that met the inclusion criteria; these papers were included in this systematic review and meta-analysis [14-29]. No additional studies were identified through the hand-searching process. The characteristics of the included studies are listed in Table 1. The details of the study population, the prevalence of AF, ambulatory devices, measuring time, and measurement data are presented in 2 respective tables for non-12-lead ECG and PPG in Multimedia Appendix 2.

**Table 1:** Characteristics of the included studies and study population.

Study authors	Year	Country	Study design	Index test	n^a^	Population
Chen et al [14]	2020	China	Prospective, cross-sectional	Non-12-lead ECG^b^ and PPG^c^	401	Inpatients and outpatients aged >18 years in a cardiovascular department
Lown et al [15]	2020	United Kingdom	Prospective, cross-sectional	Non-12-lead ECG	415	Participants aged >65 years (n=79 with AF^d^ and n=336 without AF)
Wegner et al [16]	2020	Germany	Prospective, cross-sectional	Non-12-lead ECG	92	Inpatients with no predefined exclusion criteria
Yan et al [17]	2020	Hong Kong	Prospective, cross-sectional	PPG	44	20 patients with permanent AF and 24 control individuals in sinus rhythm
Reverberi et al [18]	2019	Italy	Prospective, longitudinal	Non-12-lead ECG	95	Patients aged >18 years diagnosed with AF and scheduled for elective cardioversion
Proesmans et al [19]	2019	Belgium	Prospective, cross-sectional	Non-12-lead ECG & PPG	223	Patients aged ≥65 years, individuals with known AF and supplemented with individuals without AF
Himmelreich et al [20]	2019	Netherlands	Prospective, cross-sectional	Non-12-lead ECG	214	Patients aged ≥18 years assigned to 12-lead ECG for any nonacute indication
Haverkamp et al [21]	2019	Norway	Prospective, cross-sectional	Non-12-lead ECG	94	Patients admitted to the cardiology ward with ongoing 12-lead ECG surveillance
Fan et al [22]	2019	China	Prospective, cross-sectional	PPG	108	Patients aged ≥18 years admitted to the hospital
Yan et al [23]	2018	Hong Kong	Prospective, cross-sectional	PPG	217	Patients admitted to the cardiology ward
William et al [24]	2018	United States	Prospective, cross-sectional	Non-12-lead ECG	52	Patients aged 35-85 years diagnosed with AF and scheduled for anti-arrhythmic drug initiation
Rozen et al [25]	2018	United States	Prospective, longitudinal	PPG	98	Patients aged >18 years diagnosed with AF and scheduled for elective DC^e^ cardioversion
Bumgarner et al [26]	2018	United States	Prospective, longitudinal	Non-12-lead ECG	100	Patients aged 18-90 years diagnosed with AF and scheduled for elective cardioversion
Lown et al [27]	2018	United Kingdom	Prospective, cross-sectional	Non-12-lead ECG	418	Patients aged >65 years (n=82 with AF and n=336 without AF)
Desteghe et al [28]	2016	Belgium	Prospective, cross-sectional	Non-12-lead ECG	265	Patients aged ≥18 years admitted to the cardiology ward
Haberman et al [29]	2015	United States	Prospective, cross-sectional	Non-12-lead ECG	381	Athletes, students, and patients of an ambulatory cardiology clinic

^a^n: number of participants.

^b^ECG: electrocardiogram.

^c^PPG: photoplethysmography.

^d^AF: atrial fibrillation.

^e^DC: direct current.

### Characteristics and Quality of Studies

All the included studies followed a prospective design and had a total of 3217 participants. Of the 16 publications, 13 were cross-sectional studies [14-17,19-24,27,28], where the measurement was conducted at a single point, and the other 3 were longitudinal [18,25,26], where the measurement was conducted more than once for each participant. The sample size of the studies ranged from 44 to 418 participants. Four studies excluded participants aged <65 years, 4 studies recruited only participants with a diagnosis or history of AF [18,24-26], and 4 studies identified patients with AF and supplemented the cohort with control participants [15,17,19,27]. The remaining 7 studies recruited in-hospital patients with various indications [14,16,20-23,28], and 1 study recruited athletes, students, and patients of an ambulatory cardiology clinic [29]. The AF prevalence among the participants in these studies ranged from 5% to 100%.

The 16 studies investigated 2 technologies: non-12-lead ECG and PPG. This review defines non-12-lead ECG as measurements recorded by any electrode-based device; participants simply placed their fingers on the electrodes, attached the electrodes to their chest, or held the electrodes in their hands. PPG is a technology that measures changes in tissue blood volume that enables the recording of each heartbeat; the signal can be detected by any device with a camera monitoring various body parts, including the fingertip, wrist, palm, and face. Among the publications included in this review, 10 studies focused on non-12-lead ECG equipped in 10 different electrode-based ambulatory devices [15,16,18,20,21,24,26-29]; 4 studies focused on PPG recorded by cameras [17,23], phone cameras [19,22,23,25], or wristbands [14,22]; and 2 studies investigated both technologies in the same cohort [14,19]. All measurements were processed automatically using algorithms or smartphone apps. The reference standard in all the studies was 12-lead-ECG, which was interpreted by competent physicians or cardiologists in a blinded manner. Data for the primary statistical analysis, including true positives, false positives, false negatives, and true negatives, are presented in Multimedia Appendix 2.

**Figure 2:**
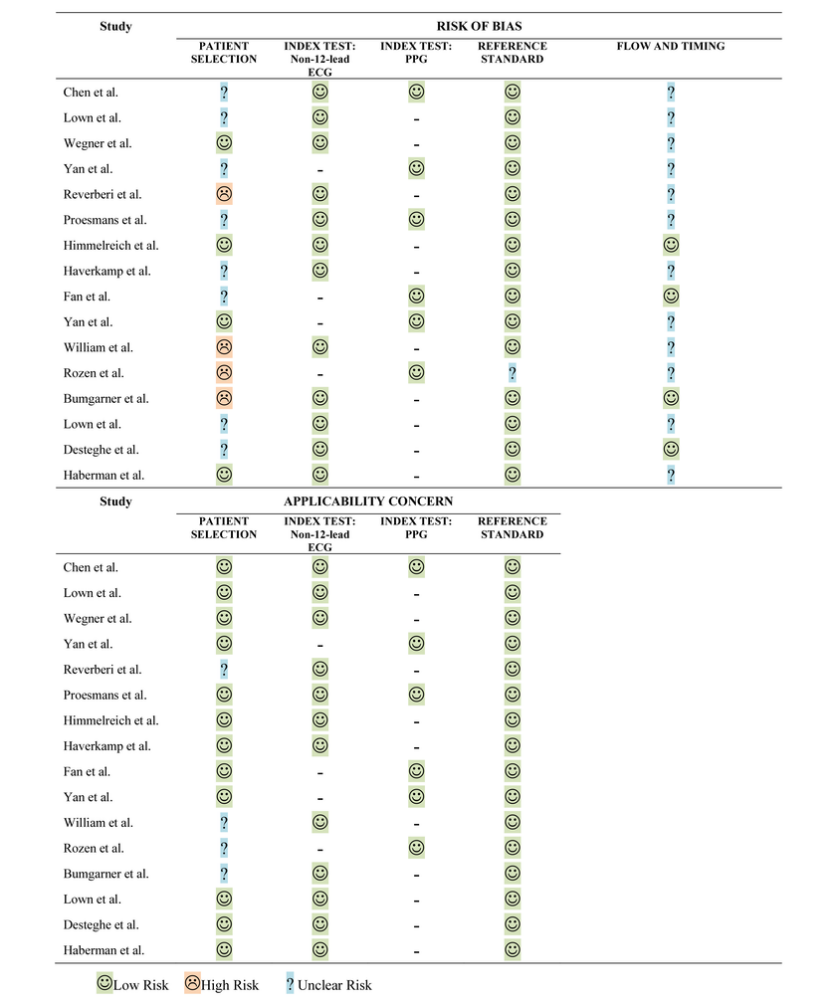
Summary of the QUADAS-2 quality appraisal of the included studies. ECG: electrocardiogram; PPG: photoplethysmography.

The quality of the included studies was evaluated according to the criteria of the QUADAS-2 tool. Regarding the risk of patient selection bias, case-control studies and participants from cardiology departments were labelled as unclear risk in yellow, and studies that included only patients with AF were labelled as high risk in red. To be included in this review, all studies must have used 12-lead-ECGs as the reference standard; however, if studies used other types of reference tests in a small proportion of participants, they were still included but marked as unclear risk in the reference standard column. In the flow and timing assessment, studies that sequentially performed the reference test right before or after the index test were labelled as unclear risk, whereas studies that performed both index and reference tests simultaneously on the participants were labelled as low risk. Other criteria were assessed as provided. Two independent reviewers performed the quality appraisal, and the results are presented in Figure 2.

### Data Synthesis of Diagnostic Accuracy

In total, 3217 participants with 7623 measurements were included in the data synthesis. 

**Figure 3:**
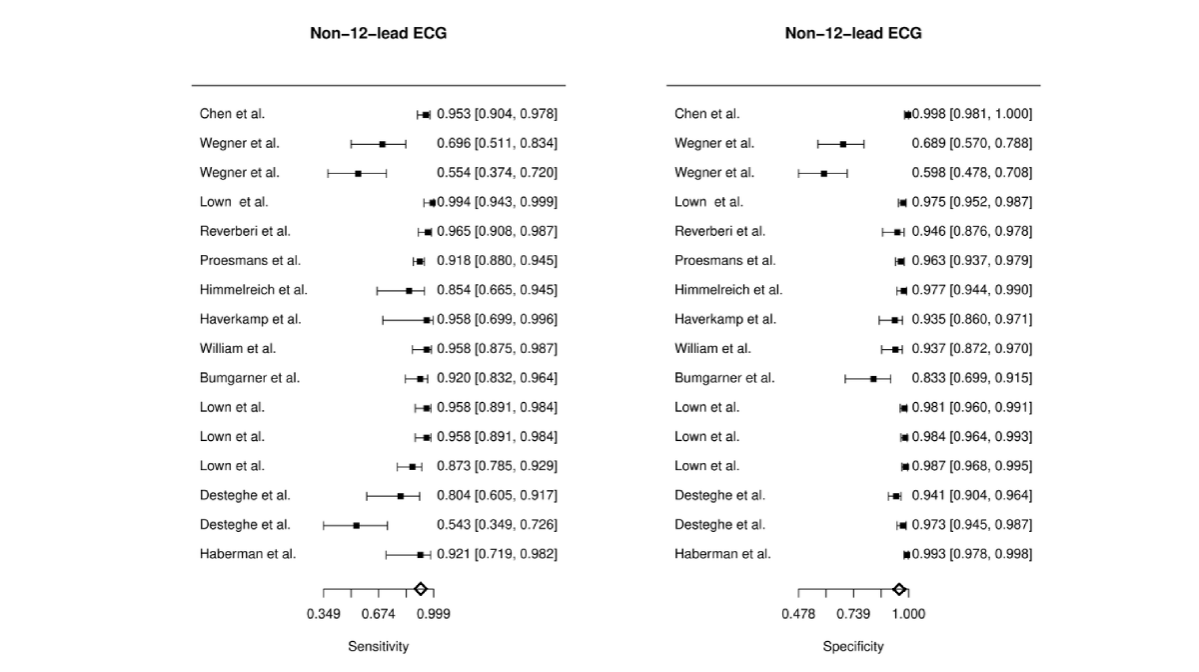
Forest plot of the combined diagnostic estimates of sensitivity and specificity of automatically interpreted non-12-lead electrocardiograms (ECGs).

**Figure 4:**
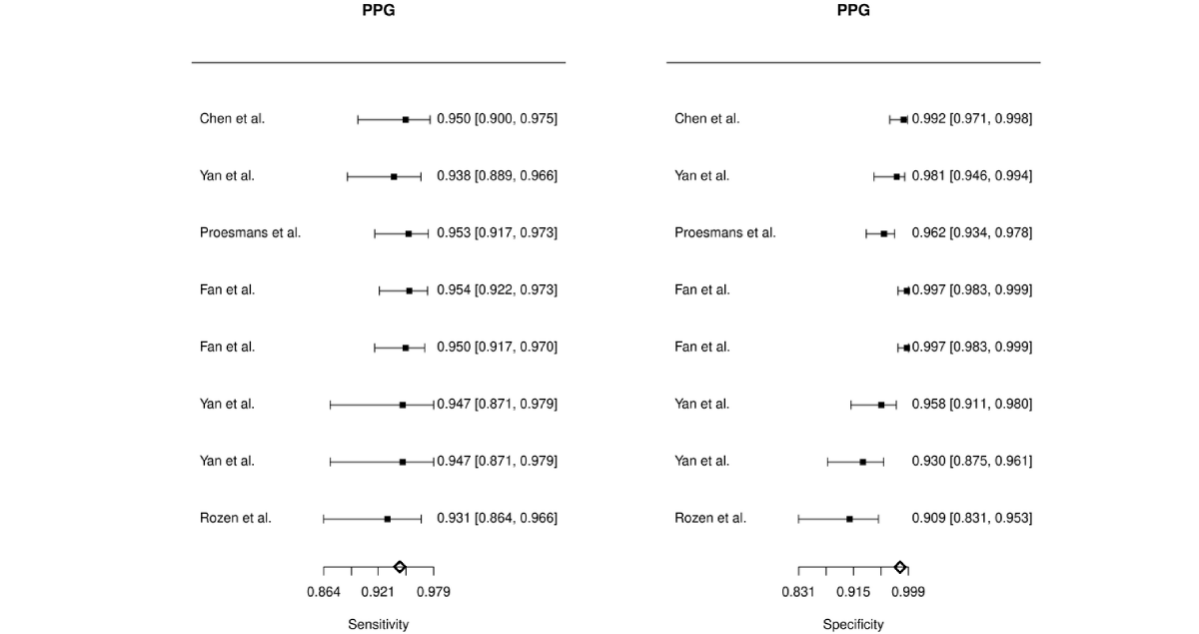
Forest plot of the combined diagnostic estimates of sensitivity and specificity of automatically interpreted photoplethymography (PPG).

**Figure 5:**
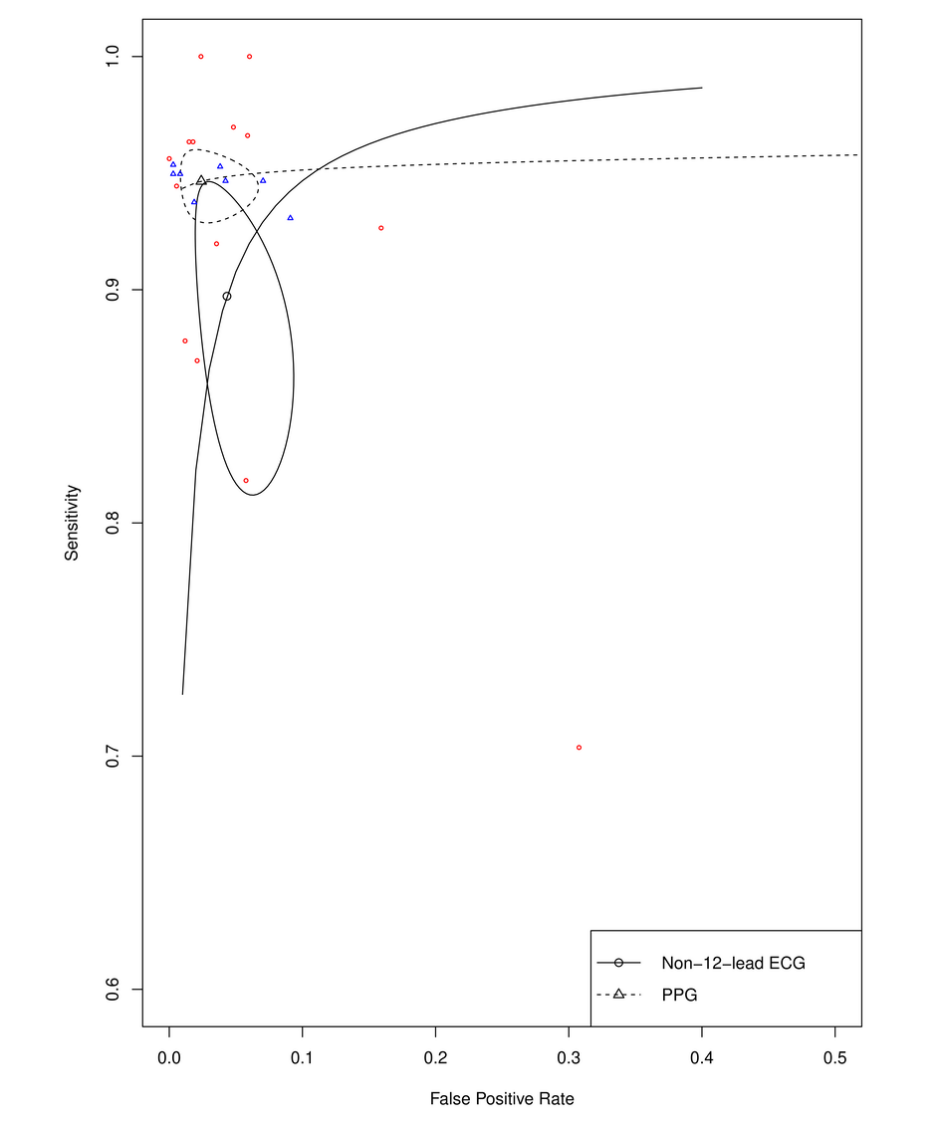
Summary receiver operating curves of the automatically interpreted non-12-lead electrocardiogram (ECG) and photoplethymography (PPG) in the diagnosis of atrial fibrillation.

Automatic detection of AF based on non-12-lead ECG had a combined estimated sensitivity of 89.7% (95% CI 83.2%-93.9%), specificity of 95.7% (95% CI 92.0%-97.7%), PLR of 20.64 (95% CI 10.10-42.15), NLR of 0.11 (95% CI 0.06-0.19), and DOR of 224.75 (95% CI 70.10-720.56). A heterogeneity test among included studies was assessed with a Cochran Q of 13.99 (df=15, *P*=.526) and Higgins I^2^ of 0%. Automatic detection of AF based on recordings of PPG had a combined estimated sensitivity of 94.7% (95% CI 93.3%-95.8%), specificity of 97.6% (95% CI 94.5%-99.0%), PLR of 35.51 (95% CI 18.19-69.31), NLR of 0.05 (95% CI 0.04-0.07), and DOR of 730.79 (95% CI 309.33-1726.49). A test of heterogeneity for the PPG studies reported a Cochran Q of 8.78 (df=7, *P*=.269) and Higgins I^2^ of 20.26%. The forest plots of the pooled diagnostic accuracies are presented in Figure 3 and Figure 4. SROC curves for both the technologies in all the included studies are presented in Figure 5. The AUSROCs were 0.97 for non-12-lead ECG and 0.95 for PPG.

**Figure 6:**
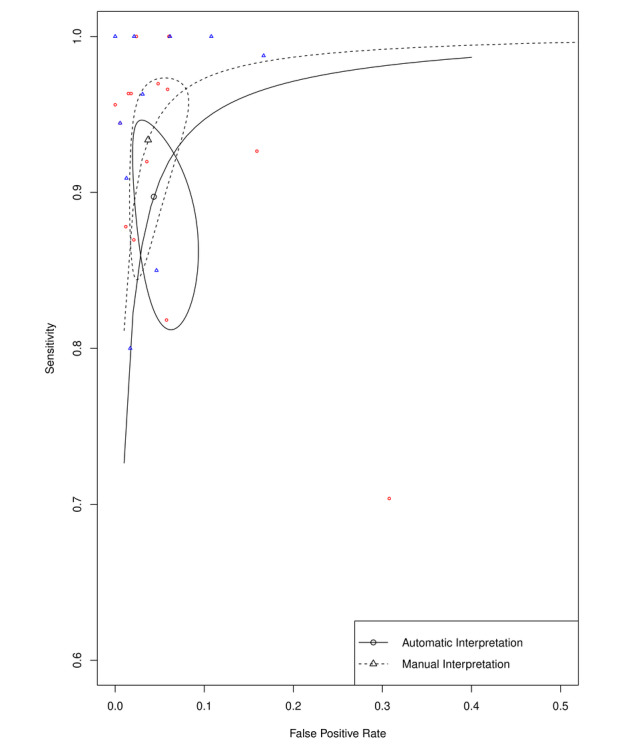
Summary receiver operating curves of automatic and manual interpretations of non-12-lead electrocardiogram in the diagnosis of atrial fibrillation.

In light of the 2020 European Society of Cardiology Clinical Practice Guidelines for the diagnosis and management of AF [30] that recommended a manually interpreted, single-lead ECG ≥30 seconds as the other option to establish a definitive diagnosis of AF, we also extracted relevant data from the included studies to perform a meta-analysis of such a method. Manual interpretation based on non-12-lead ECG had a combined sensitivity of 93.4% (95% CI 86.7%-96.8%), specificity of 96.3% (95% CI 92.9%-98.1%), PLR of 25.93 (95% CI 13.70-49.05), NLR of 0.07 (95% CI 0.04-0.14), and DOR of 439.64 (95% CI 202.89-952.65). All non-12-lead ECG segments included were recorded ≥30 seconds. The forest plots of the pooled diagnostic accuracies are shown in Multimedia Appendix 3. An SROC comparison curve between the automatic and manual interpretations of non-12-lead ECG are presented in Figure 6. No PPG recordings were examined manually among the included studies.

### Subgroup Analysis: Study Population

**Figure 7:**
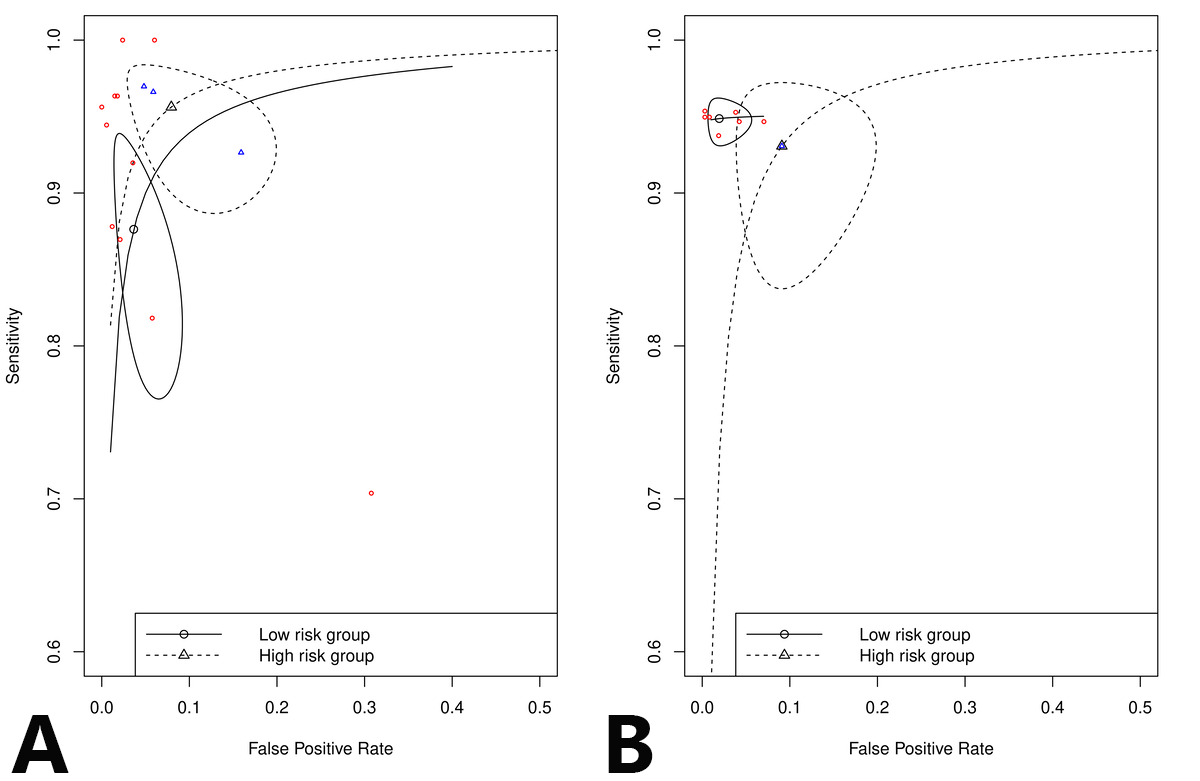
Subgroup analysis of the study population, including a comparison of summary receiver operating curves between the low-risk study population, which included patients with and without atrial fibrillation, and high-risk study population, which included only patients with atrial fibrillation, for (A) non-12-lead electrocardiogram and (B) photoplethysmography.

A subgroup analysis (Figure 7) was performed to investigate the effect of the study design and population on the diagnostic accuracy. Studies were divided into 2 groups: low risk (group 1), including those that recruited participants with and without AF, and high risk (group 2), including those that only recruited participants with prediagnosed AF.

Non-12-lead ECG yielded a sensitivity of 87.6% and specificity of 96.4% in low-risk studies (n=13) and a sensitivity of 95.6% and specificity of 92.1% in high-risk studies (n=3). PPG yielded a sensitivity of 94.9% and specificity of 98.1% in low-risk studies (n=7) and a sensitivity of 93.1% and specificity of 90.9% in high-risk studies (n=1). The subgroup analysis of non-12-lead ECG indicated a higher sensitivity and lower specificity among the high-risk studies. On the other hand, only 1 study was included in the high-risk group for PPG; thus, no conclusion could be made regarding the subgroup analysis of the PPG technology.

### Fagan Nomogram

The Fagan nomogram analysis (Multimedia Appendix 4 and Multimedia Appendix 5) demonstrated that, with an age-adjusted prevalence of 0.5% in the general population with pooled PLR of 20.64 and pooled NLR of 0.11, the posttest probabilities of non-12-lead ECG increased to 9.40% and decreased to 0.06%, respectively; with pooled PLR of 35.51 and pooled NLR of 0.05, the posttest probabilities of PPG increased to 15.14% and decreased to 0.03%, respectively. By contrast, in a high-risk population with a prevalence of 2.3%, the application of non-12-lead ECG had posttest probabilities of 32.70% and 0.26%, respectively, and PPG had posttest probabilities of 45.53% and 0.12%, respectively.

### Publication Bias

Publication bias was assessed using Deek funnel plots (Multimedia Appendix 6 and Multimedia Appendix 7). For studies investigating the non-12-lead ECG method, visual inspection of the funnel plot indicated a likely absence of publication bias (*P*=.107). For studies investigating the PPG method, a visual evaluation of the funnel plot also provided no clear evidence of publication bias (*P*=.107).

## Discussion

### Principal Findings

This systematic review and meta-analysis on the diagnostic accuracy of 16 studies published between 2015 and 2021 confirmed that both non-12-lead ECG and PPG are highly accurate technologies in detecting AF. Automatically interpreted PPG provided the highest sensitivity and specificity for the diagnosis of AF, immediately followed by the manual interpretation of non-12-lead ECG, whereas automatically diagnosed AF based on non-12-lead ECG performed relatively weaker than the first 2 methods. However, they all demonstrated outstanding diagnostic accuracy. The differences among these approaches may be attributed to measuring techniques: PPG optically records volumetric changes in local arterioles and, in this manner, measures pulse variability (or R-R interval) and heart rhythm variability, while non-12-lead ECG assesses the electrical activity of the heart using electrodes. Other possible explanations for the variability include different classification thresholds and different methods used in the algorithm design.

Early diagnosis of AF is crucial for preventing AF-related morbidity, mortality, and economic burden. The prevalence of undiagnosed AF in the United States is estimated to be 1%-2%, and the incremental cost burden could amount to US $3.1 billion per year [31]. In comparison, smart wearable devices and mobile phones are almost ubiquitous worldwide, and the incremental cost for their application is relatively low. Our summarized findings suggest that the appropriate application of these technologies enables early diagnosis and treatment, which might possibly contribute to reducing the cost of illness. The current guidance suggests pulse palpation and 12-lead ECG in AF screening. However, in a meta-analysis by Taggar et al [6], pulse palpation was proved inferior to 4 other methods because of lower specificity; additionally, the use of 12-lead ECG is limited by its inconvenience and the fact that it captures an ECG at only 1 time point. In clinical practice, continuous Holter monitoring is one of the most commonly used methods to detect AF. However, this examination requires patients to carry the machine with them for an extended period, which results in inconvenience; thus, it is also not a suitable tool for general screening.

The 2 main technologies reviewed in this meta-analysis, non-12-lead ECG and PPG, have several advantages as screening tools for AF. First, they involve lightweight, low-cost, and ambulatory devices. In this review, non-12-lead ECGs were employed in devices such as handheld tablets [16,19-21,24,27,28], watchbands [14,26], handheld rod-like sensors [28], and electrodes attached to the chest [15,16,18,19,27]. By contrast, PPG signals can be assessed on the fingertips [19,22,23,25], wrist [14,22], earlobe, and face [17,23] by any device with simple optoelectronic components, such as a smartphone. Second, these technologies require a relatively short monitoring time, which enables fast and timely screening; the monitoring time most commonly varied from 30 seconds to 1 minute. Third, measurements obtained by these devices were automatically interpreted by built-in algorithms, which means that the tests can be conducted without the presence of a health care professional. Several studies [16,20,24,26,28] included in this review conducted additional analyses comparing algorithms’ and physicians’ interpretations of the same non-12-lead ECG segments and concluded that automated algorithm performance is not inferior to competent professional interpretation. Finally, as demonstrated in the results, the automatically generated diagnoses established with both technologies yielded outstanding diagnostic accuracy. Automatically interpreted PPG had a sensitivity and specificity of 94.7% and 97.6%, respectively. Automatically interpreted non-12-lead ECG had a sensitivity and specificity of 89.7% and 95.7%, respectively. As for the interpretation of non-12-lead ECG by competent physicians, an established method to diagnose AF according to the 2020 European Society of Cardiology Clinical Guidelines had a sensitivity and specificity of 93.4% and 96.3%, respectively. Demonstrating effectiveness, convenience, and time savings with high diagnostic accuracies, we suggest using these technologies with built-in automatic interpretation as preliminary screening tools for the detection of AF when the gold standard method, which generally requires a physician’s interpretation, is not feasible. Notably, the screening program should target high-risk populations (eg, elderly) to avoid false positives.

We performed a subgroup analysis of both technologies on account of the apparent patient selection bias among included studies. The sensitivity and specificity of a test are generally believed to not vary with the disease prevalence of a population; however, variations of these diagnostic parameters were often spotted. According to a previous article that summarized the phenomenon and proposed several possible causes [32], the specificity of a test tended to be lower with high disease prevalence, and although not significant, the sensitivity appeared higher with high prevalence of some diseases. This review also observed instability of the sensitivity and specificity between low-risk groups (studies that recruited patients with and without AF) and high-risk groups (studies that recruited only patients with AF). Higher sensitivity and lower specificity were generated in the high-risk groups using the non-12-lead ECG tests; however, no conclusion could be made for the PPG method because only 1 study included a high-risk group. The results indicate that the performance of these technologies was affected by the recruited population and design of the included studies. Future validation conducted in a more general population is warranted to investigate such an occurrence.

### Strengths and Limitations

To our knowledge, this is the first systematic review and meta-analysis to compare the diagnostic accuracy of 2 common technologies, namely non-12-lead ECG and PPG, used in ambulatory devices for detecting AF. This review followed PRISMA guidelines, implemented a comprehensive search strategy, applied strict inclusion and exclusion criteria, and employed 2 independent reviewers to assess all the included studies. For instance, one of the criteria was to exclude studies that failed to provide a timely reference standard; this resulted in the exclusion of multiple large-scale screening studies but ensured time consistency between the index test and reference standard. Moreover, we investigated the heterogeneity and publication bias of included studies, as well as the posttest probability of AF using these ambulatory device technologies. Finally, an additional subgroup analysis was performed to investigate the effect of the study population on diagnostic performance. The result identified sensitivity and specificity variations between low-risk and high-risk populations, indicating future validation of the diagnostic accuracy of these tests is needed in a more general population.

This review of studies on AF detection using ambulatory devices has several limitations. The most noteworthy concern is the study population investigated. Except for the 4 studies that recruited only patients with AF for their assessment, other studies were mostly conducted in a case-control style or recruited inpatients from hospitals. This problem was reflected in the QUADAS-2 quality assessment and possibly contributed to the instability of diagnostic accuracy in the subgroup analysis. Additionally, the heterogeneity of devices and algorithms used should be considered. Although we explored the accuracy of ambulatory devices from a technology perspective, these technologies were applied in diverse devices, and the measurements were automatically interpreted by their respective algorithms. Furthermore, measurements of insufficient quality or unclassified by algorithms tended to be excluded in the calculation of the diagnostic accuracy in some of the studies [14,19,22,24,26]; the proportion of insufficient or unclassified recordings ranged from 0.5% to 33.8%. Finally, some studies regarded atrial flutter as the same disease state as AF, viewing the incident as a positive AF result [16,19,20,24,26-29].

### Conclusions

Both non-12-lead ECG and PPG technologies offered high diagnostic accuracies for AF. Automatically interpreted PPG recordings generated the highest sensitivity and specificity compared to both the manual and automatic interpretations of non-12-lead ECG. Detection of AF employing automatic analysis techniques may serve as a useful preliminary screening tool before administering a gold standard test, which generally requires analyses by competent physicians. Subgroup analysis indicated variations of sensitivity and specificity between studies that recruited low-risk and high-risk populations, and future validation of these diagnostic tests in the general population is warranted.

## Appendix

Multimedia Appendix 1 The literature search strategy for the systematic review and meta-analysis in Medline (Ovid) and Embase databases.

Multimedia Appendix 2 Details of the study population, ambulatory devices, measuring time, reference standard, prevalence, and measurement data of the included studies.

Multimedia Appendix 3 Forest plot of the combined diagnostic estimates of sensitivity and specificity of manually interpreted non-12-lead electrocardiogram.

Multimedia Appendix 4 Fagan nomogram for the evaluation of the clinical utility of automatically interpreted non-12-lead electrocardiogram (ECG).

Multimedia Appendix 5 Fagan nomogram for the evaluation of the clinical utility of automatically interpreted photoplethysmography (PPG).

Multimedia Appendix 6 Deek funnel plots for the assessment of publication bias of non-12-lead electrocardiogram (ECG) studies.

Multimedia Appendix 7 Deek funnel plots for the assessment of publication bias of photoplethysmography (PPG) studies.

## Abbreviations

AF: atrial fibrillation
AUSROC: area under the summary receiver operating curve
DOR: diagnostic odds ratio
ECG: electrocardiogram
NLR: negative likelihood ratio
PLR: positive likelihood ratio
PPG: photoplethysmography
PRISMA: Preferred Reporting Items for Systematic Reviews and Meta-Analyses
QUADAS: quality assessment of diagnostic accuracy studies
SROC: summary receiver operating characteristic
