# Supplementary material for: Diagnostic Accuracy of Ambulatory Devices in Detecting Atrial Fibrillation: Systematic Review and Meta-analysis
Source: JMIR Mhealth Uhealth. 2021 Apr 9;9(4):e26167. doi: 10.2196/26167 (PMC8065566; doi:10.2196/26167)
Supplement: Multimedia Appendix 1 [file mhealth_v9i4e26167_app1.docx]

| Database: Medline (Ovid) | | Results | Database: Embase | | Results |
| --- | --- | --- | --- | --- | --- |
| 1 | atrial fibrillation.mp. or Atrial Fibrillation/ | 86947 | 1 | ‘atrial fibrillation’/exp OR ‘atrial fibrillation’ | 179123 |
| 2 | mobile application.mp. or Mobile Applications/ | 7984 | 2 | ‘mobile phone’/exp OR ‘mobile phone’ | 34081 |
| 3 | Wearable Electronic Devices/ or wearable.mp. | 15277 | 3 | ‘mobile application’/exp OR ‘mobile application’ | 14619 |
| 4 | mhealth.mp. or Telemedicine/ | 29655 | 4 | wearable | 16710 |
| 5 | digital treatment.mp. | 58 | 5 | ‘mhealth’/exp OR ‘mhealth’ | 6323 |
| 6 | telemedicine.mp. or Telemedicine/ | 33019 | 6 | ‘telemedicine’/exp OR ‘telemedicine’ | 54437 |
| 7 | 2 or 3 or 4 or 5 or 6 | 56415 | 7 | digital AND (‘treatment’/exp OR ‘treatment’) | 50424 |
| 8 | 1 and 7 | 418 | 8 | #2 OR #3 OR #4 OR #5 OR #6 OR #7 | 158281 |
| 9 | limit 8 to yr=“2015-Current” | 335 | 9 | #7 AND #8 | 1624 |
| 10 | limit 9 to english | 325 | 10 | #9 AND ([adult]/lim OR [aged]/lim OR [middle aged]/lim OR [very elderly]/lim OR [young adult]/lim) AND (2015:py OR 2016:py OR 2017:py OR 2018:py OR 2019:py OR 2020:py OR 2021:py) AND [english]/lim | 750 |
| 11 | limit 10 to “all adult (19 plus years)” | 114 |  |  |  |
