# Supplementary material for: Diagnostic Accuracy of Ambulatory Devices in Detecting Atrial Fibrillation: Systematic Review and Meta-analysis
Source: JMIR Mhealth Uhealth. 2021 Apr 9;9(4):e26167. doi: 10.2196/26167 (PMC8065566; doi:10.2196/26167)
Supplement: Multimedia Appendix 2 [file mhealth_v9i4e26167_app2.docx]

| Study & Year | Study design | n | Mean age | Technology & Device | Measuring time | Reference standard | TP | FP | FN | TN | P |
| --- | --- | --- | --- | --- | --- | --- | --- | --- | --- | --- | --- |
| Chen et al., 2020 | Cross-sectional | 401 | 64 | Single-channel wristband ECG | 60 sec | 12-lead-ECG | 131 | 0 | 6 | 249 | 37.4% |
| Lown et al., 2020 | Cross-sectional | 415 | NA | Chest electrodes ECG | 60 consecutive beats | 12-lead-ECG | 79 | 8 | 0 | 328 | 19.0% |
| Wegner et al., 2020 | Cross-sectional | 92 | 64 | Single-lead ECG | 30 sec | 12-lead-ECG | 19 | 20 | 8 | 45 | 29.4% |
| Wegner et al., 2020 | Cross-sectional | 92 | 64 | Parasternal lead ECG | 30 sec | 12-lead-ECG | 15 | 26 | 12 | 39 | 29.4% |
| Reverberi et al., 2019 | Longitudinal | 95 | 66 | Chest belt ECG | 10 min | 12-lead-ECG | 96 | 4 | 3 | 79 | 100% |
| Proesmans et al., 2019 | Cross-sectional | 210 | 67 | Single-lead Chest electrodes ECG | 1 min | 12-lead-ECG | 252 | 12 | 22 | 326 | 64.6% |
| Himmelreich et al., 2019 | Cross-sectional | 214 | 64 | Single-lead ECG | 30 sec | 12-lead-ECG | 20 | 4 | 3 | 187 | 10.7% |
| Haverkamp et al., 2019 | Cross-sectional | 94 | 58 | Single-lead ECG | 30 sec | 12-lead-ECG | 11 | 5 | 0 | 78 | 11.7% |
| William et al., 2018 | Cross-sectional | 52 | 68 | Single-lead ECG | 30 sec | 12-lead-ECG | 57 | 6 | 2 | 96 | 100% |
| Bumgarner et al., 2018 | Longitudinal | 100 | 68 | Watchband Single-lead ECG | 30 sec | 12-lead-ECG | 63 | 7 | 5 | 37 | 92.0% |
| Lown et al., 2018 | Cross-sectional | 418 | 74 | Chest electrodes ECG | 60 consecutive beats | 12-lead-ECG | 79 | 6 | 3 | 330 | 19.6% |
| Lown et al., 2018 | Cross-sectional | 418 | 74 | Electrode-based ECG | 60 consecutive beats | 12-lead-ECG | 79 | 5 | 3 | 331 | 19.6% |
| Lown et al., 2018 | Cross-sectional | 418 | 74 | Single-lead ECG | 30 sec | 12-lead-ECG | 72 | 4 | 10 | 332 | 19.6% |
| Desteghe et al., 2016 | Cross-sectional | 265 | 70 | Rod-like single-lead ECG | 30 sec | 12-lead-ECG | 18 | 14 | 4 | 229 | 35.6% |
| Desteghe et al., 2016 | Cross-sectional | 265 | 70 | Single-lead ECG | 30 sec | 12-lead-ECG | 12 | 6 | 10 | 237 | 35.6% |
| Haberman et al., 2015 | Cross-sectional | 381 | 35 | Single-lead ECG | 30 sec | 12-lead-ECG | 17 | 2 | 1 | 361 | 4.5% |

*Abbreviations*: *ECG* electrocardiogram, *n* number of participants, *TP* true positive, *FP* false positive*, FN* false negative*, TN* true negative, *P* prevalence, *NA* not available

| Study & Year | Study design | n | Mean age | Technology & Device | Measuring time | Reference standard | TP | FP | FN | TN | P |
| --- | --- | --- | --- | --- | --- | --- | --- | --- | --- | --- | --- |
| Chen et al., 2020 | Cross-sectional | 401 | 64 | Wristband PPG | 3 min | 12-lead-ECG | 132 | 2 | 7 | 242 | 37.4% |
| Yan et al., 2020 | Cross-sectional | 44 | 66 | Facial PPG | 1 min | 12-lead-ECG | 150 | 3 | 10 | 157 | 45.5% |
| Proesmans et al., 2019 | Cross-sectional | 207 | 77 | Fingertip PPG | 1 min | 12-lead-ECG | 222 | 12 | 11 | 302 | 64.6% |
| Fan et al., 2019 | Cross-sectional | 108 | 62 | Wrist PPG | 3 min | 12-lead-ECG | 267 | 1 | 13 | 333 | 48.1% |
| Fan et al., 2019 | Cross-sectional | 108 | 62 | Fingertip PPG | 3 min | 12-lead-ECG | 264 | 1 | 14 | 332 | 48.1% |
| Yan et al., 2018 | Cross-sectional | 217 | 70 | Facial PPG | 1 min | 12-lead-ECG | 71 | 6 | 4 | 136 | 34.6% |
| Yan et al., 2018 | Cross-sectional | 217 | 70 | Fingertip PPG | 1 min | 12-lead-ECG | 71 | 10 | 4 | 132 | 34.6% |
| Rozen et al., 2018 | Longitudinal | 98 | 68 | Fingertip PPG | 20 sec (*3) | 12-lead-ECG | 94 | 8 | 7 | 80 | 100% |

*Abbreviations*: *PPG* plethymography, *n* number of participants, *TP* true positive, *FP* false positive*, FN* false negative*, TN* true negative, *P* prevalence, *NA* not available

*These figures were not provided directly and were calculated on the basis of available data
