# Supplementary figures and images for: Diagnostic Accuracy of Ambulatory Devices in Detecting Atrial Fibrillation: Systematic Review and Meta-analysis
Source: JMIR Mhealth Uhealth. 2021 Apr 9;9(4):e26167. doi: 10.2196/26167 (PMC8065566; doi:10.2196/26167)

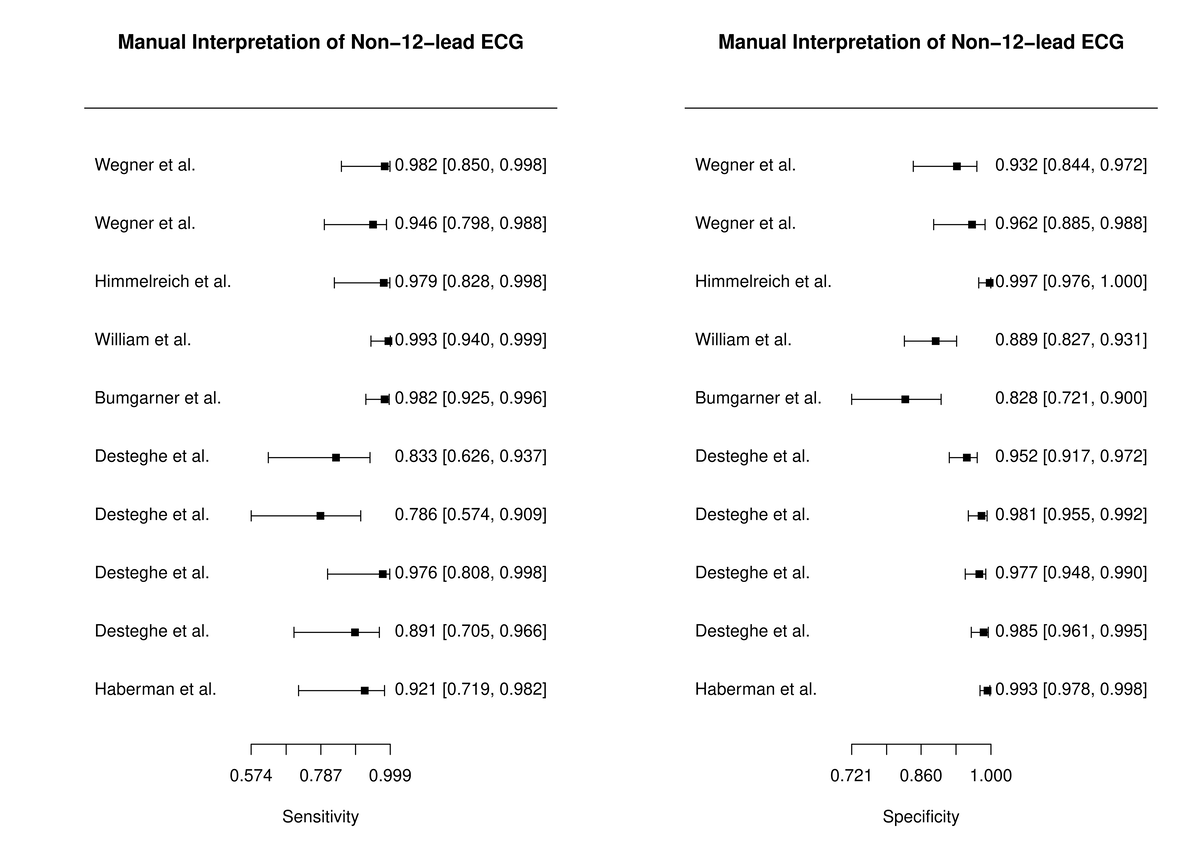

Supplement: Multimedia Appendix 3 [file mhealth_v9i4e26167_app3.png]

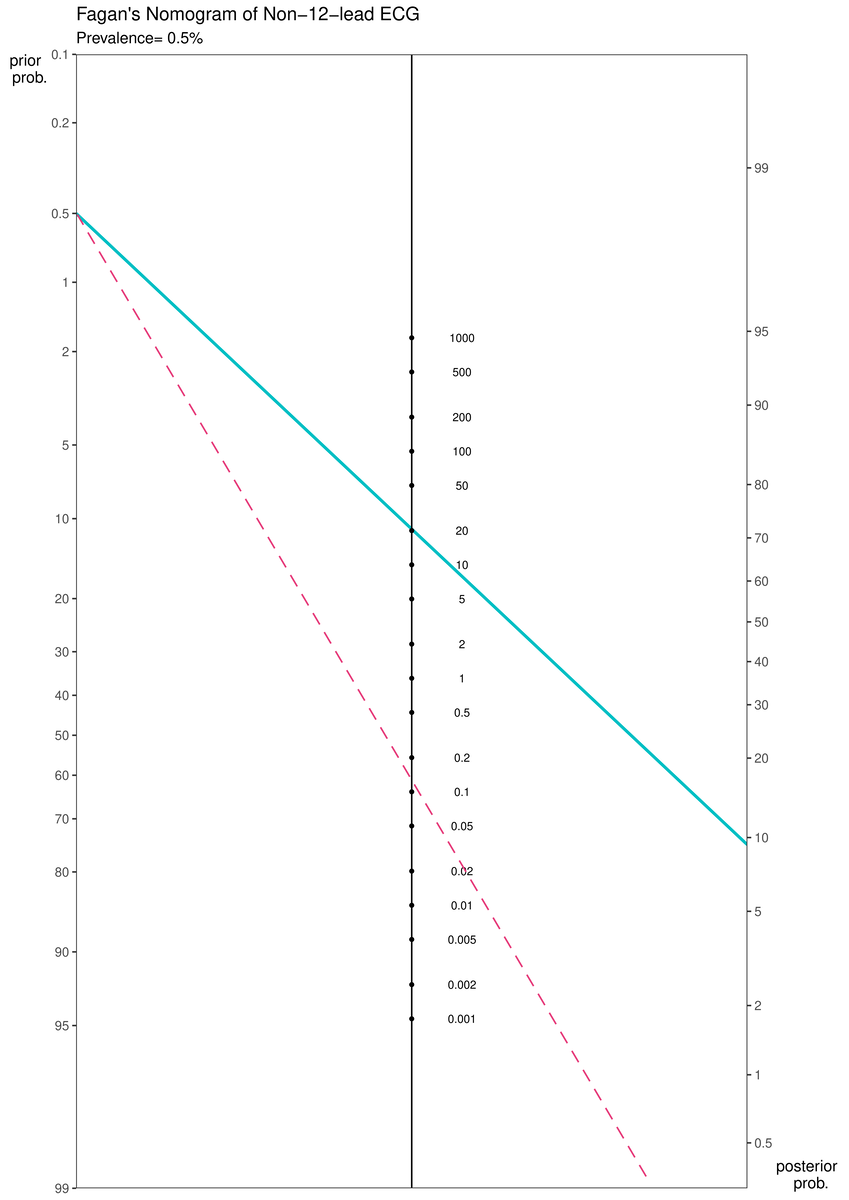

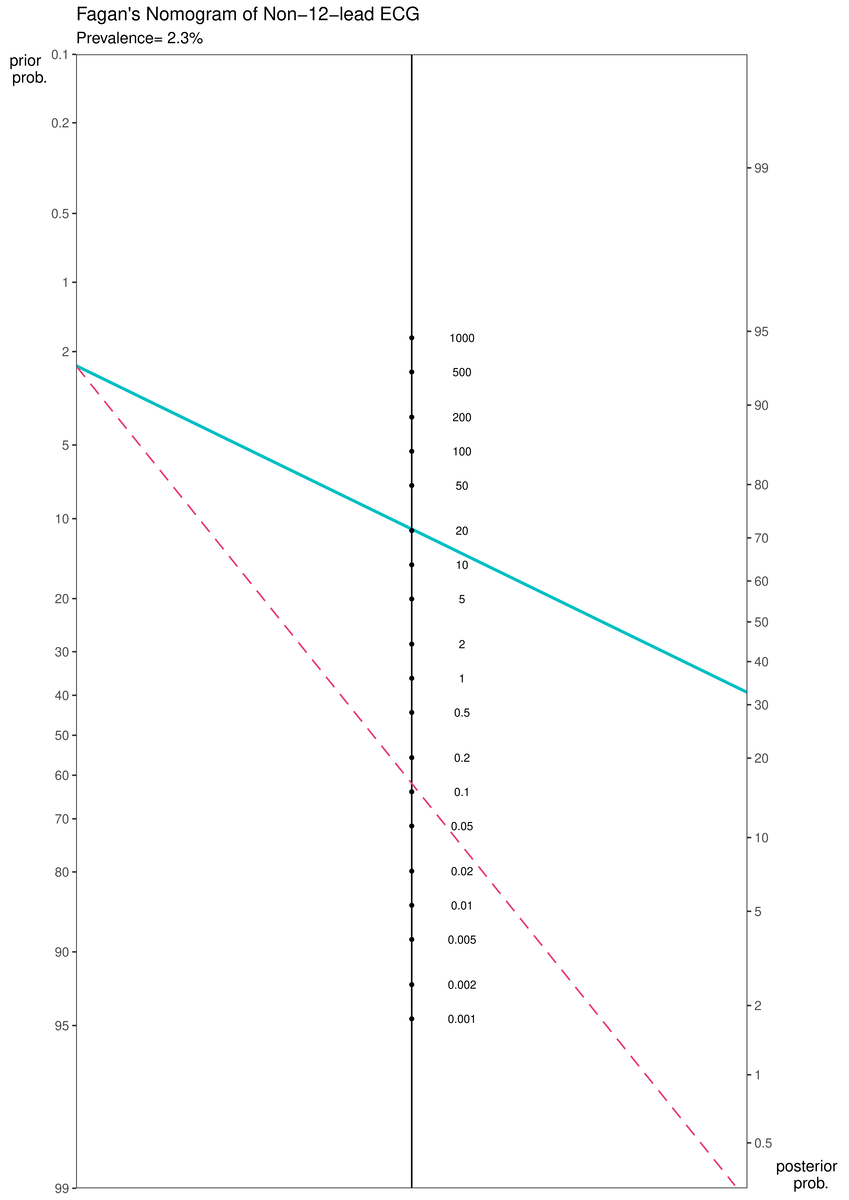

Supplement: Multimedia Appendix 4 [file mhealth_v9i4e26167_app4.docx]

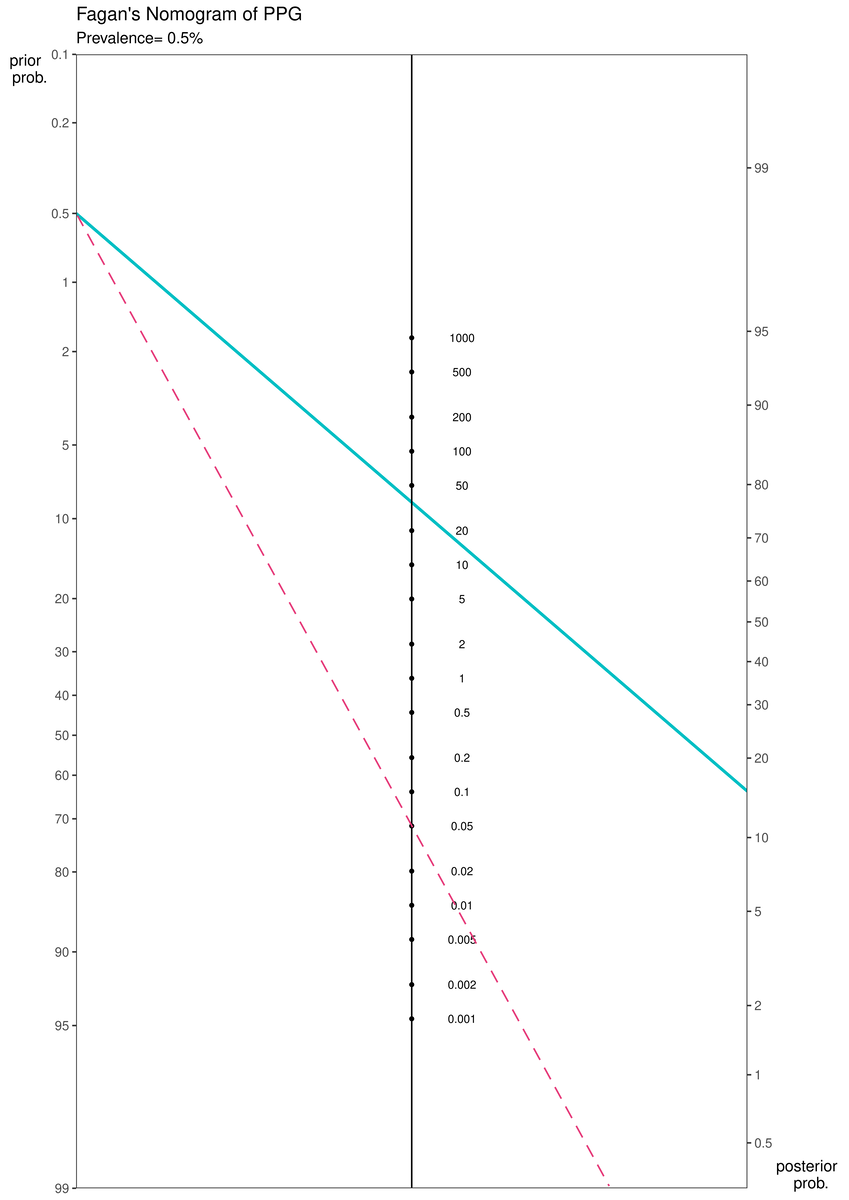

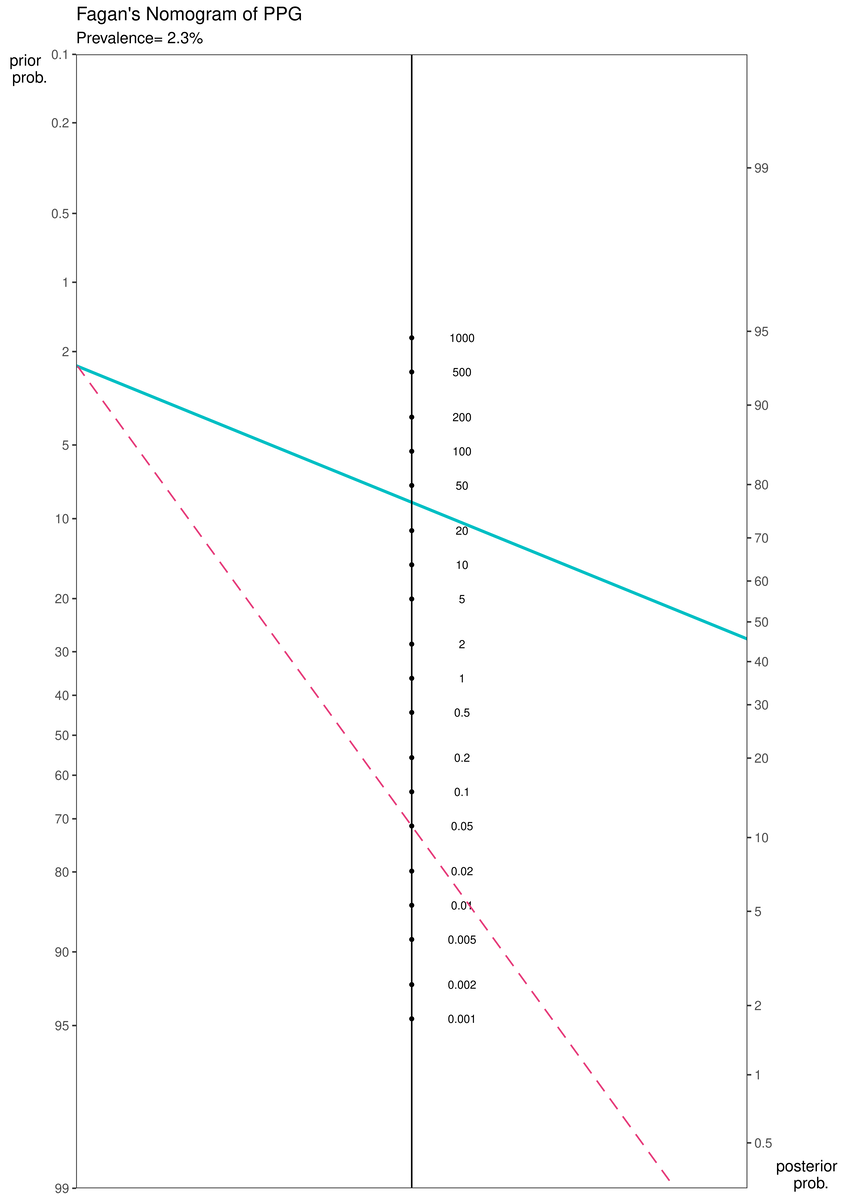

Supplement: Multimedia Appendix 5 [file mhealth_v9i4e26167_app5.docx]

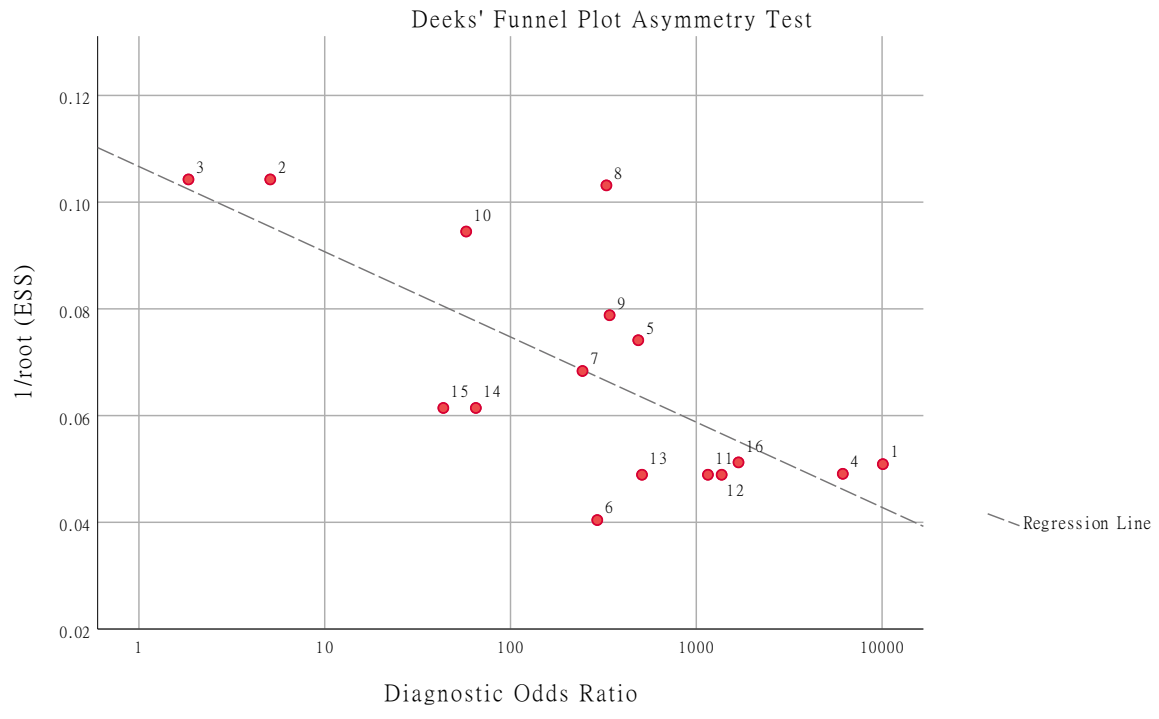

Supplement: Multimedia Appendix 6 [file mhealth_v9i4e26167_app6.pdf]

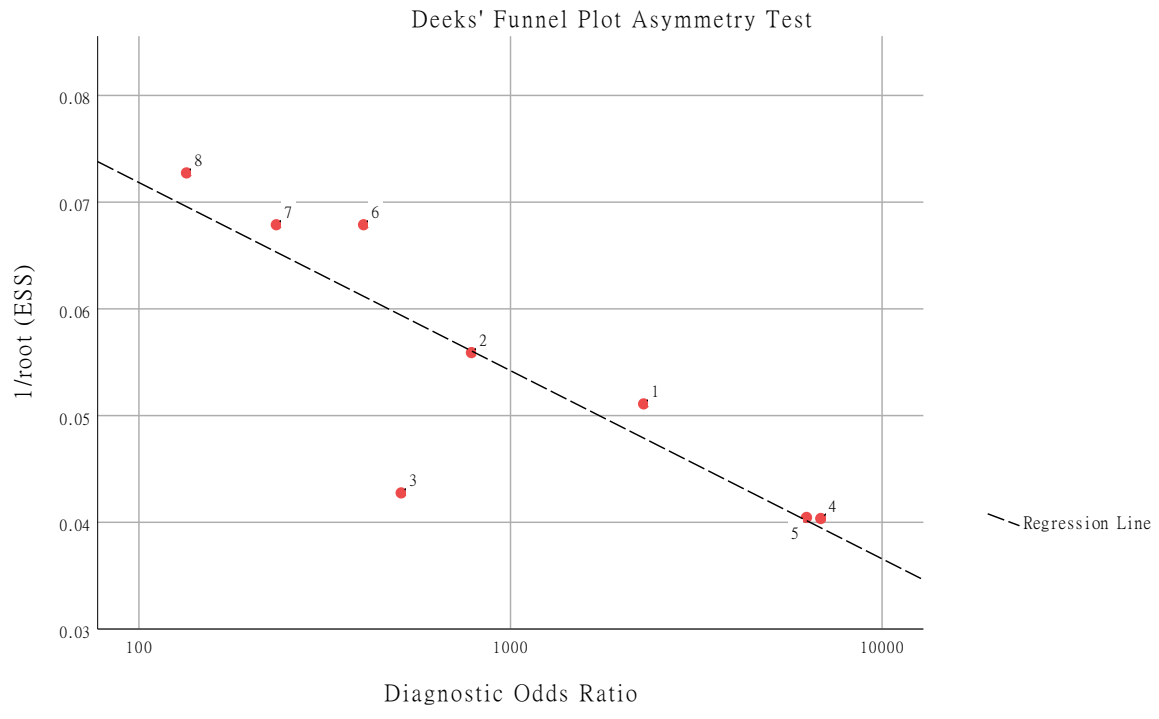

Supplement: Multimedia Appendix 7 [file mhealth_v9i4e26167_app7.pdf]
